# Optimization of Dissolved Air Flotation in the Treatment of Coffee Processing Wastewater Using Calcium Chloride

**DOI:** 10.1021/acsomega.5c06919

**Published:** 2025-10-30

**Authors:** Ana Carolina Chaves Dourado, Alisson Carraro Borges

**Affiliations:** Department of Agricultural Engineering, 28120 Federal University of Viçosa, Av. P.H. Rolfs, s/n, Viçosa, MG 36570-900, Brazil

## Abstract

Coffee processing wastewater (CPW) is characterized by a high organic load, low pH, and elevated solids, and its improper disposal can cause severe environmental impacts. Coagulation-flocculation followed by dissolved air flotation (DAF) has proven effective for CPW treatment. This study evaluated CaCl_2_ as a clarifier and the optimization of DAF operational parameters using a central composite rotational design. With CaCl_2_, removals of 65% turbidity and 70% UV_254_ were achieved, while treatment without the salt reached only 40% turbidity and no significant UV_254_ reduction. The optimal conditions with CaCl_2_ were a 5 bar saturation pressure, an 80% recirculation ratio, and a 30 min flotation time; without salt, similar pressures and recirculation ratios were obtained, but the flotation time increased to 36 min. These findings highlight the role of CaCl_2_ in enhancing the DAF performance for CPW treatment.

## Graphical abstract

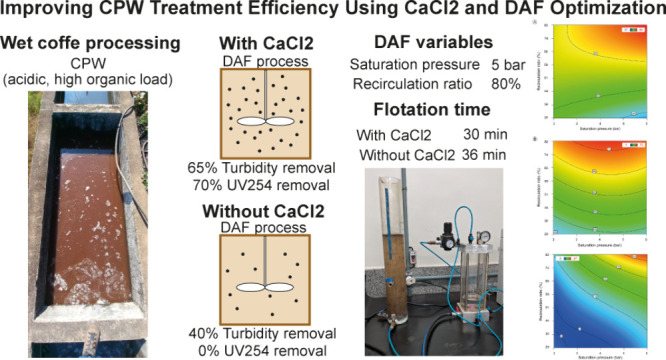

## 1 Introduction

Coffee is the second most traded agricultural commodity in the world, second only to oil, and is among the most important products in international trade. The main producing and exporting countries include Brazil, Colombia, Indonesia, Honduras, and Ethiopia.^1^ During harvesting, coffee beans are sent for separation and processing, which can be carried out using the dry or wet method, which utilizes specific techniques depending on the maturity and quality of the beans.^2^

On a global scale, the wet method is the most widely used method in coffee production and is widely recognized for providing higher quality coffee compared to the dry method. However, despite its processing efficiency, this method generates a large volume of effluent, known as coffee processing wastewater (CPW). CPW has low pH, high organic load, high concentration of nutrients, and high levels of suspended and dissolved solids.^3^ The coffee processing sector is one of the agricultural industries that pollutes the environment the most, and improper disposal of CPW can cause environmental imbalances, necessitating proper treatment before discharging it into receiving water bodies.^4^

Physicochemical treatments, such as coagulation-flocculation, are widely used in wastewater treatment because of their effectiveness in removing solids, organic matter, and other pollutants. In addition, this technique is an alternative with an advantageous cost–benefit ratio.^5^

Coagulation-flocculation is used to bind colloidal particles into larger aggregates, reducing water turbidity and removing other organic and inorganic substances. This process involves two distinct stages: first, rapid mixing of the coagulant into the wastewater by means of intense agitation and then the flocculation stage, which consists of gently agitating the mixture to form well-defined flocs that bring together smaller particles. In this method, chemical agents are added to wastewater to destabilize the organic colloidal suspensions, facilitating their separation after the process.^5^

Salinity can improve the coagulation-flocculation process because salts, such as calcium chloride, increase the ionic strength and help neutralize the charges of the suspended particles, which facilitates the formation of flocs.^6^

After coagulation-flocculation, the flocs formed can be separated using dissolved air flotation (DAF).^7^ Dissolved air flotation is a technique used in wastewater treatment in which microbubbles are generated to adhere to suspended particles. The aim of this process is to remove the suspended particles, causing the agglomerates to rise to the surface and promoting clarification. In DAF, microbubbles are introduced directly into the treatment.^8^

The quantity of microbubbles generated in a dissolved air flotation system is one of the most important operational variables in the process. This bubble quantity is influenced by the saturation pressure and recirculation ratio. The flotation time also plays an essential role, as an appropriate period is required for effective collision between the flocs and microbubbles.^9^ DAF is considered a more effective technique than sedimentation for treating wastewater with low-density particles. This process can result in better-quality wastewater.^9^

A study carried out by Dourado et al.^10^ showed that coagulation-flocculation followed by DAF in the treatment of CPW is promising. The authors used green coagulants (moringa seeds and pitaya cladodes) extracted with calcium chloride. However, the specific role of calcium chloride in this process has not been explored alone.

Other studies have demonstrated the application of calcium chloride as an isolated clarifier in different industrial effluents. Jamali and Moradnia^11^ applied 4.2 g L^–1^ CaCl_2_ at pH 3.7 to machining fluid effluents, achieving removals of over 90% of turbidity. In another example, AlMubaddal et al.^12^ tested CaCl_2_ alone in effluents from a PVC plant, achieving removals nearly 65% for turbidity. These studies highlight the potential of CaCl_2_ as a single clarifier/coagulant in physicochemical wastewater treatment processes.

In view of this, this study focused exclusively on the use of calcium chloride as an aid in the CPW clarification process, seeking to investigate its effectiveness without interference from other products. The relevance of this research lies in the need to understand the potential of this salt in the coagulation-flocculation process followed by DAF, contributing to the development of more efficient and sustainable perspectives for the treatment of CPW.

Considering the saturation pressure, recirculation ratio, and flotation time as independent variables and the removal of turbidity and UV_254_ compounds as response variables, the aim of this study was to optimize the treatment of CPW with DAF under two conditions: the presence and absence of calcium chloride as a clarifying agent.

## 2 Materials and Methods

### 2.1 Wastewater

The coffee processing wastewater (CPW) was collected during the harvest at the “Colibri e Jatobá” farm, located in the municipality of Paula Cândido, Minas Gerais, Brazil. The samples were packed in plastic vessels and refrigerated at 4 °C in a cold room for the time of the experiment. The values obtained for characterizing the CPW are shown in Table 1. All analyses were performed in accordance with the Standard Methods for the Examination of Water and Wastewater*.* ^13^

**Table 1:** Characteristics of Coffee Processing Wastewater

**constituent (unit)**	**value**
pH	4.6
turbidity (NTU)	868
potassium (mg L^–1^)	151.5
electrical conductivity (μS cm^–1^)	1567
temperature (°C)	23.7
SUVA (L mg^–1^ m^–1^)	0.0055
total COD (mg L^–1^)	7597
filtered COD (mg L^–1^)	3207
filtered TOC (mg L^–1^)	2064
UV_254_ organic compounds	11.26
calcium (mg L^–1^)	14.0
total copper (mg L^–1^)	<0.08
total solids (mg L^–1^)	12494
total volatile solids (mg L^–1^)	11202
total fixed solids (mg L^–1^)	1292

### 2.2 Experimental Planning

In the first stage, calcium chloride was used exclusively as a coagulant, acting as a clarifying agent during the coagulation-flocculation phase without the application of other coagulants. In the second stage, the DAF operating parameters were optimized without the addition of calcium chloride or any other coagulant.

The central composite rotational design (CCRD) was based on a 2^3^ factorial involving eight factorial points, six axial points, and six center points, totaling 20 trials carried out independently and randomly. The high and low levels of the variables were determined based on preliminary tests as well as according to the limitations of the floating apparatus used in the experiment. Table 2 shows the saturation pressure, recirculation ratio, and flotation time used in the experiment, both with and without the addition of calcium chloride.

**Table 2:** Coded and Actual Levels of the Independent Variables in the CCRD Using Calcium Chloride and in the Absence of the Salt

	**levels of independent variables**				
**independent variables**	–α	–1	**0**	**+1**	**+α**
saturation pressure (bar)	2.00	2.81	4.00	5.18	6.00
recirculation ratio (%)	10.00	28.24	55.00	81.75	100.00
flotation time (min)	5.00	13.10	25.00	36.89	45.00

To calculate the removal of turbidity and UV_254_ compounds, the average values of the three readings taken for each of the 20 tests were considered to minimize errors.

To determine the optimum levels, Design Expert software was used to identify a mathematical model related to the evaluated parameters including saturation pressure, recirculation ratio, and flotation time. The models were validated by using three independent tests.

The value of α was calculated based on the number of independent variables (*k* = 3) using eq 1:

$$\alpha ={\left(2^{3}\right)}^{1/4}=1.682$$ 1

To determine the optimum points, Design Expert software was used to identify the appropriate conditions for the parameters studied, such as the saturation pressure, recirculation ratio, and flotation time, with the removal efficiency. These points represent a combination of the levels of independent variables that result in the best efficiency of the process evaluated. Finally, the models were validated in three independent runs.

### 2.3 Coagulation-Flocculation followed by DAF

In the coagulation-flocculation process, two separate experiments were carried out: first, only calcium chloride was added as an aid to the coagulation process to assess its effectiveness in destabilizing the colloids; second, calcium chloride was not added in order to compare the results without the use of this coagulant in the process.

The fast and slow mixing stages were performed in a jar test (model 218-2 LDB, Ethik Technology), and the mixing speeds and times were specific based on preliminary tests. The mixing conditions were the same for both the experiments.

In each test, 1 L of CPW was added to 2 L beakers with rapid stirring at 400 rpm for 1 min. This was followed by rapid mixing and stirring at 400 rpm for 1 min.^14^ At this stage, in the first experiment, 1.5 g of calcium chloride (CaCl_2_, analytical grade, 99–107% purity, Synth; manufacturer: Êxodo Científica) was used, as indicated by Dourado et al.^10^ It should be noted that the dosage of 1.5 g of calcium chloride was determined based on preliminary tests, in which this concentration proved to be the minimum necessary to enable the coagulation-flocculation process and achieve efficient removal of turbidity and UV_254_-absorbing compounds, which are the response variables in this study.

After coagulation, flocculation began, and agitation was reduced to 150 rpm for 15 min.^14^ It should be noted that calcium chloride was not used in the second experiment, but the fast and slow stirring conditions were the same as those used in the first experiment. In both cases, the pH was not adjusted and the natural pH of CPW was maintained at 4.6. After the flocculation stage, the equipment was switched off and the contents of the beaker were slowly transferred to the flotation column to preserve the flocs.

In the dissolved air flotation (DAF) stage, the equipment called “flotatest” (218-3 Flow, Nova Ética) was used to separate the flocs formed during the coagulation-flocculation process. This equipment consists of a compressor, a saturation chamber, and a flotation column and was adapted by Pereira et al.^15^

The parameters studied in the process were the saturation pressure (bar), recirculation ratio (%), and flotation time (min), which were determined and optimized using statistical planning.

At the start of the process, 2 L of tap water was placed in the saturation chamber. The compressor was then switched on to inject air into the chamber, and the pressure was adjusted according to the values limited by the equipment (2–6 bar). During all tests, the saturation chamber was pressurized for 2 min before opening the valve to inject saturated water into the flotation column. The recirculation ratio was determined using different values (10–100%) of the 2 L volume in the saturation chamber. When the values were reached, according to the experimental plan, the valve was closed and the flotation time was waited, which varied between 5 and 45 min. Finally, CPW (0.5 L) was collected from the bottom of the flotation column to analyze the turbidity and UV_254_ compounds.

pH values of raw and treated CPW did not differ significantly with the addition of calcium chloride. Thus, the zeta potential of raw wastewater and wastewater treated with calcium chloride was determined before and after the coagulation step using a Litesizer 500 particle analyzer (Anton Paar, Austria) via electrophoretic mobility. Measurements were performed in triplicate under different pH conditions to evaluate the influence of the salt and acidification on the colloidal stability of the effluent.

### 2.4 Analytical Determinations

Analyses of the response variables (turbidity and UV_254_ compounds) were performed in accordance with the guidelines of the Standard Methods for the Examination of Water and Wastewater*.* ^13^ All variables were measured before and after CPW treatment to determine the removal efficiency of the parameters.

The turbidity was analyzed by using a portable turbidity meter (ORION AQ3010). To measure the UV_254_ compounds, the treated sample was filtered using glass microfiber filters with a pore size of 1.00 μm and a diameter of 45 mm. The sample was read on a spectrophotometer (model Hach DR 6000) set to a wavelength of 254 nm by using a 1 cm quartz cuvette. Equation 2 was used to determine absorbance at 254 nm.

$${\mathrm{UV}}_{254}=\frac{A}{b}\times D$$ 2

where UV_254_ is the UV absorbance in cm^–1^; *b* is the optical path in cm, *A* corresponds to the average absorbance measured, and *D* is the dilution factor.

To calculate the removal efficiency (ε) of the variables (turbidity and UV_254_), eq 3 proposed by Couto et al.^16^ was used. To calculate the absolute removal of these parameters, eq 4, as recommended by Muniz,^17^ was used.

$$\varepsilon =\left(1-\frac{XV}{X_{0}V_{0}}\right)\times 100$$ 3

$$R=X_{0}V_{0}-XV$$ 4

where ε represents the removal efficiency and is expressed as a percentage (%). *R* corresponds to the removal of turbidity (NTU) and UV_254_ compounds (cm^–1^) at the end of CPW treatment with calcium chloride. For the tests carried out without the addition of salt, the calculation applies only to the turbidity. *V* corresponds to the volume of CPW (L) at the end of the process, considering that the volume recirculated during flotation was adjusted according to the experimental design. *X*_0_ refers to turbidity and UV_254_ compounds at the start of the treatment, while *V*_0_ indicates the initial CPW volume (L). To calculate the removal of turbidity and UV_254_ compounds, the average values of the three readings taken for each of the 20 tests were considered to minimize errors.

## 3 Results and Discussion

### 3.1 Calcium Chloride Tests

#### 3.1.1 Results of the Experimental Runs

Table 3 shows the data for the removal efficiency of turbidity and UV_254_ compounds using calcium chloride in the process. It can be seen that the turbidity removal varied between 30.74 and 68.89%. Regarding UV_254_ compounds, removal ranged from 4.80 to 76.02%. All of the runs were randomized.

**Table 3:** Removal Efficiency Values Using Calcium Chloride in the Process

**tests**	**pressure (bar)**	**recirculation ratio (%)**	**flotation time (min)**	**turbidity removal efficiency (%)**	**UV**_**254**_ **compound removal efficiency (%)**
1	3	30	13	38.99	4.80
2	5	30	13	45.07	14.74
3	3	80	13	35.00	53.82
4	5	80	13	64.65	58.44
5	3	30	37	49.12	11.90
6	5	30	37	33.46	19.01
7	3	80	37	62.26	65.36
8	5	80	37	65.55	72.29
9	2	55	25	56.21	34.72
10	6	55	25	62.50	42.18
11	4	10	25	30.74	17.94
12	4	100	25	68.89	76.02
13	4	55	5	58.15	32.86
14	4	55	45	53.79	51.51
15	4	55	25	55.48	55.24
16	4	55	25	58.39	51.51
17	4	55	25	52.58	58.97
18	4	55	25	54.76	49.64
19	4	55	25	64.44	44.05
20	4	55	25	62.26	53.37

Table 4 shows the removal efficiency for turbidity without the use of calcium chloride. It should be noted that an experiment was also conducted without the addition of salt to assess the removal of UV_254_ compounds; however, no significant removal of these compounds was observed under these conditions. Turbidity removal efficiency ranged from no removal to 46.77%. Runs were performed randomly.

**Table 4:** Removal Efficiency Values without the Addition of Calcium Chloride

**tests**	**pressure (bar)**	**recirculation ratio (%)**	**flotation time (min)**	**turbidity removal efficiency (%)**
1	3	30	13	3.04
2	5	30	13	5.81
3	3	80	13	8.34
4	5	80	13	12.24
5	3	30	37	5.07
6	5	30	37	6.91
7	3	80	37	11.34
8	5	80	37	36.80
9	2	55	25	no removal
10	6	55	25	9.27
11	4	10	25	4.19
12	4	100	25	46.77
13	4	55	5	2.98
14	4	55	45	18.47
15	4	55	25	10.73
16	4	55	25	9.76
17	4	55	25	6.13
18	4	55	25	10.48
19	4	55	25	6.85
20	4	55	25	5.40

#### 3.1.2 Statistical Analysis and Regression Model

The models were selected based on the best *R* ^2^, adjusted *R* ^2^, predictive *R* ^2^, and adequate precision indicators. The same criterion was applied to choose the presentation of the response variables (percentage removal, remaining value, or removed value), and the percentage removal of turbidity and UV_254_ compounds was identified as the best responses obtained.

The second-order mathematical models describing the removal of turbidity and UV_254_ compounds using calcium chloride as a function of the saturation pressure, recirculation ratio, and flotation time are shown in eqs 5 and 6, respectively.

$$\mathrm{turbidity}\;\mathrm{removal}\left(\mathrm{NTU}\right)=20.1111+3.5309\times \mathrm{saturation}\;\mathrm{pressure}-0.02785\times \mathrm{recirculation}\;\mathrm{ratio}+1.1799\times \mathrm{flotation}\;\mathrm{time}+0.1670\times \mathrm{saturation}\;\mathrm{pressure}\times \mathrm{recirculation}\;\mathrm{ratio}-0.4252\times \mathrm{saturation}\;\mathrm{pressure}\times \mathrm{flotation}\;\mathrm{time}+0.0116\times \mathrm{recirculation}\;\mathrm{ratio}\times \mathrm{flotation}\;\mathrm{time}-0.0053\times \mathrm{recirculation}\;{\mathrm{ratio}}^{2}$$ 5

$$\mathrm{removal}\;\mathrm{of}\;{\mathrm{UV}}_{254}\;\mathrm{compounds}\left({\mathrm{cm}}^{-1}\right)=-95.9270+33.6095\times \mathrm{saturation}\;\mathrm{pressure}+0.8131\times \mathrm{recirculation}\;\mathrm{ratio}+1.8955\times \mathrm{flotation}\;\mathrm{time}-3.8845\times \mathrm{saturation}\;{\mathrm{pressure}}^{2}-0.295\times \mathrm{flotation}\;{\mathrm{time}}^{2}$$ 6

Equation 7 shows the final third-order model developed with real variables for turbidity removal without the addition of calcium chloride. The model considers the saturation pressure, recirculation ratio, and flotation time as factors.

$$\mathrm{turbidity}\;\mathrm{removal}\left(\mathrm{NTU}\right)=132.3237-60.4200\times \mathrm{saturation}\;\mathrm{pressure}-2.7455\times \mathrm{recirculation}\;\mathrm{ratio}+0.4896\times \mathrm{flotation}\;\mathrm{time}+1.0774\times \mathrm{saturation}\;\mathrm{pressure}\times \mathrm{recirculation}\;\mathrm{ratio}-0.2701\times \mathrm{saturation}\;\mathrm{pressure}\times \mathrm{flotation}\;\mathrm{time}-0.0244\times \mathrm{recirculation}\;\mathrm{ratio}\times \mathrm{flotation}\;\mathrm{time}+8.1906\times \mathrm{saturation}\;{\mathrm{pressure}}^{2}+0.0085\times \mathrm{recirculation}\;{\mathrm{ratio}}^{2}+0.0099\times \mathrm{saturation}\;\mathrm{pressure}\times \mathrm{recirculation}\;\mathrm{ratio}\times \mathrm{flotation}\;\mathrm{time}-0.1503\times s\mathrm{aturation}\;{\mathrm{pressure}}^{2}\times \mathrm{recirculation}\;\mathrm{ratio}$$ 7

Table 5 shows the values of the coefficients of determination, which indicate how well the model fits the observed data for turbidity and UV_254_ compounds using calcium chloride. The values of the coefficients of determination (*R* ^2^, adjusted *R* ^2^, and predicted *R* ^2^) provided an important analysis of the quality of the fitted model.

**Table 5:** Indicators of Model Fit Using Calcium Chloride

**indicator**	**turbidity**	**UV**_**254**_ **compounds**
*R* ^2^	0.84	0.93
adjusted *R* ^2^ (*R* ^2^ adj)	0.75	0.91
predicted *R* ^2^ (*R* ^2^ pred)	0.30	0.84
adequate precision	11.22	22.41

With regard to turbidity, the discrepancy between the adjusted *R* ^2^ (0.75) and predicted *R* ^2^ (0.30) suggests possible problems, such as the inclusion of irrelevant variables or interactions in the experimental data. However, for the UV_254_ compounds, the adjusted *R* ^2^ of 0.91 is in good agreement with the predicted *R* ^2^ of 0.84, considering that the difference between them is less than 0.2.

An *R* ^2^ greater than 0.80 indicates greater reliability for predictive purposes, representing a more accurate model with lower errors.^18^ This behavior was observed both for turbidity, which had an *R* ^2^ of 0.84, and for UV_254_ compounds, which had an *R* ^2^ of 0.93, reinforcing the quality of the adjustments in these cases. Adequate precision greater than four represents an adequate signal, which shows adequate discrimination of the model.^19^

Table 6 shows the data from the final third-order model adjusted for turbidity removal without the use of calcium chloride. The predicted *R* ^2^ value was 0.64, while the adjusted *R* ^2^ value was 0.94, showing a difference of more than 0.2. This discrepancy can be attributed to the simplification of the model, transformation of the responses, or presence of discrepant values. Despite this, the model showed a good initial fit, explaining 97% of the variability in the data, which suggests that the equation adequately represented the points observed.

**Table 6:** Indicators of Fit of the Adjusted Model in the Absence of Calcium Chloride

**indicator**	**value**
*R* ^2^	0.97
adjusted *R* ^2^ (*R* ^2^ adj)	0.94
predicted *R* ^2^ (*R* ^2^ pred)	0.64
adequate precision	21.27

To evaluate the model, it is essential to consider the adjusted *R* ^2^, which indicates the percentage of variation in the response explained by the model by adjusting the number of predictors in relation to the total number of observations. In this study, the adjusted *R* ^2^ value was high, reflecting a good fit. However, predicted *R* ^2^ is more appropriate for assessing the model’s ability to predict responses in new observations.^15^

Tables 7 and 8 show the analysis of variance (ANOVA) of the models selected for the removal of turbidity and UV_254_ compounds, respectively, by using calcium chloride. Table 9 shows the ANOVA results of the model proposed for removing turbidity without the use of calcium chloride, considering different conditions of the saturation pressure, recirculation ratio, and flotation time. It can be seen that all of the regression models are significant (*p* *≤* 0.05) for the variations found. The lack of model fit was not significant (*p* > 0.05), indicating that the models fit the data well. The *p* ≤ 0.001 for the models of UV_254_ compounds and turbidity in the absence of calcium reinforces the good fit to the experimental data, demonstrating that the lower this value is, the better the model fit is.

**Table 7:** Analysis of Variance (ANOVA) of the Regression Model for Turbidity Removal Using Calcium Chloride

source	sum of squares	DoF	mean square	*F*-value	*p*-value	degree of significance
Model	2097.15	7	299.59	9.33	0.0005	significant
A-pressure	84.36	1	84.36	2.63	0.1311	
B-recirculation ratio	1144.13	1	1144.13	35.61	<0.0001	
C-time	27.44	1	27.44	0.8541	0.3736	
AB	226.15	1	226.15	7.04	0.0211	
CA	289.33	1	289.33	9.01	0.0110	
BC	109.75	1	109.75	3.42	0.0893	
B^2^	215.98	1	215.98	6.72	0.0235	
Residue	385.52	12	32.13			
lack of fit	279.62	7	39.95	1.89	0.2514	not significant
pure error	105.91	5	21.18			
Total	2482.67	19				

**Table 8:** Analysis of Variance (ANOVA) of the Regression Model for the Removal of UV_254_ Compounds Using Calcium Chloride

**source**	**sum of squares**	**DoF**	**mean square**	*F*-value	*p*-value	**degree of significance**
Model	7567.10	5	1513.42	39.47	<0.0001	significant
A-pressure	123.95	1	123.95	3.23	0.0938	
B-recirculation ratio	6465.41	1	6465.41	168.62	<0.0001	
C-time	339.91	1	339.91	8.86	0.0100	
A^2^	439.25	1	439.25	11.46	0.0044	
C^2^	253.67	1	253.67	6.62	0.0221	
Residue	536.82	14	38.34			
lack of fit	406.96	9	45.22	1.74	0.2808	not significant
pure error	129.86	5	25.97			
Total	8103.91	19				

**Table 9:** Analysis of Variance (ANOVA) of the Regression Model for Turbidity Removal in the Absence of Calcium Chloride

**source**	**sum of squares**	**G.L.**	**mean square**	*F*-value	*p*-value	**degree of significance**
Model	2994.95	10	299.49	31.05	<0.0001	significant
A-pressure	499.06	1	499.06	51.74	<0.0001	
B-recirculation ratio	906.56	1	906.56	93.99	<0.0001	
C-time	113.66	1	113.66	11.78	0.0075	
AB	121.31	1	121.31	12.58	0.0063	
AC	120.95	1	120.95	12.54	0.0063	
BC	185.97	1	185.97	19.28	0.0017	
A^2^	0.1794	1	0.1794	0.0186	0.8945	
B^2^	539.88	1	539.88	55.97	<0.0001	
ABC	112.50	1	112.50	11.66	0.0077	
A^2^B	107.26	1	107.26	11.12	0.0087	
Residue	86.81	9	9.65			
lack of fit	51.01	4	12.75	1.78	0.2696	not significant
pure error	35.79	5	7.16			
Total	3081.76	19				

#### 3.1.3 Data Validation

During the validation stage, three additional runs were performed to validate the models. The results of these three conditions were validated by comparing the observed values to those estimated using the models. The aim of this stage was to verify that the average of the observed values was within the 95% prediction interval (PI), as shown in Tables 10 and 11.

**Table 10:** Validation Results for Turbidity and UV_254_ Compounds Using Calcium Chloride

**variables**	**values**		
Saturation pressure (bar)	5	3	5
Recirculation ratio (%)	80	40	70
Flotation time (min)	30	15	30
Turbidity removal (NTU)			
observed value	56.70	47.77	60.21
estimated value	64.09	46.61	64.09
lower 95% PI	50.58	32.51	60.71
upper 95% PI	77.61	60.71	77.61
Removal of UV_254_ compounds			
observed value	74.00	22.86	60.00
estimated value	62.22	24.25	62.22
lower 95% PI	47.95	9.85	47.95
upper 95% PI	76.50	38.66	76.50

**Table 11:** Validation Results for Turbidity in the Absence of Calcium Chloride

**variables**			**turbidity removal (NTU)**			
**saturation pressure (bar)**	**recirculation ratio (%)**	**flotation time (min)**	**observed value**	**estimated value**	**lower 95% PI**	**upper 95% PI**
5	80	36	36.68	45.44	35.92	54.96
3	40	15	9.07	2.06	–6.01	10.15
5	70	30	31.61	25.27	17.56	32.98

Table 10 shows the results of the validation of the removal of turbidity and UV_254_ compounds using calcium chloride, while Table 11 illustrates the removal of turbidity without the use of this salt.

It should be noted that the models were adequately validated for both the tests with calcium chloride and those without calcium chloride, confirming that the average of the observed results is within the 95% prediction interval (95% PI). These results prove the accuracy and reliability of the models, showing that their estimates were adjusted to the experimental data, which make them safe for use.

#### 3.1.4 Determining the Optimum Regions Considering the Response Variables with and without the Use of Calcium Chloride

Contour plots enable the assessment of the influence of important operating parameters on the performance of the DAF applied to the treatment of CPW. The variables analyzed included the saturation pressure, recirculation ratio, and flotation time as well as a direct comparison between the use and absence of calcium chloride in the coagulation-flocculation process.

Figure 1 shows the results obtained with the addition of calcium chloride. Figure 1A shows the removal of turbidity with a maximum efficiency of 65%, whereas Figure 1B shows the removal of UV_254_ compounds, achieving an efficiency of up to 70%. On the other hand, Figure 2 shows turbidity removal without the use of calcium chloride, showing lower efficiency, with a maximum value of 40%. These results emphasize the importance of calcium chloride in the process, enhancing the removal of turbidity and the organic compounds represented by UV_254_. In addition, they make it possible to identify the ideal operating conditions, focusing on the parameters of saturation pressure and recirculation ratio, to optimize the efficiency of the CPW treatment.

**Figure 1:**
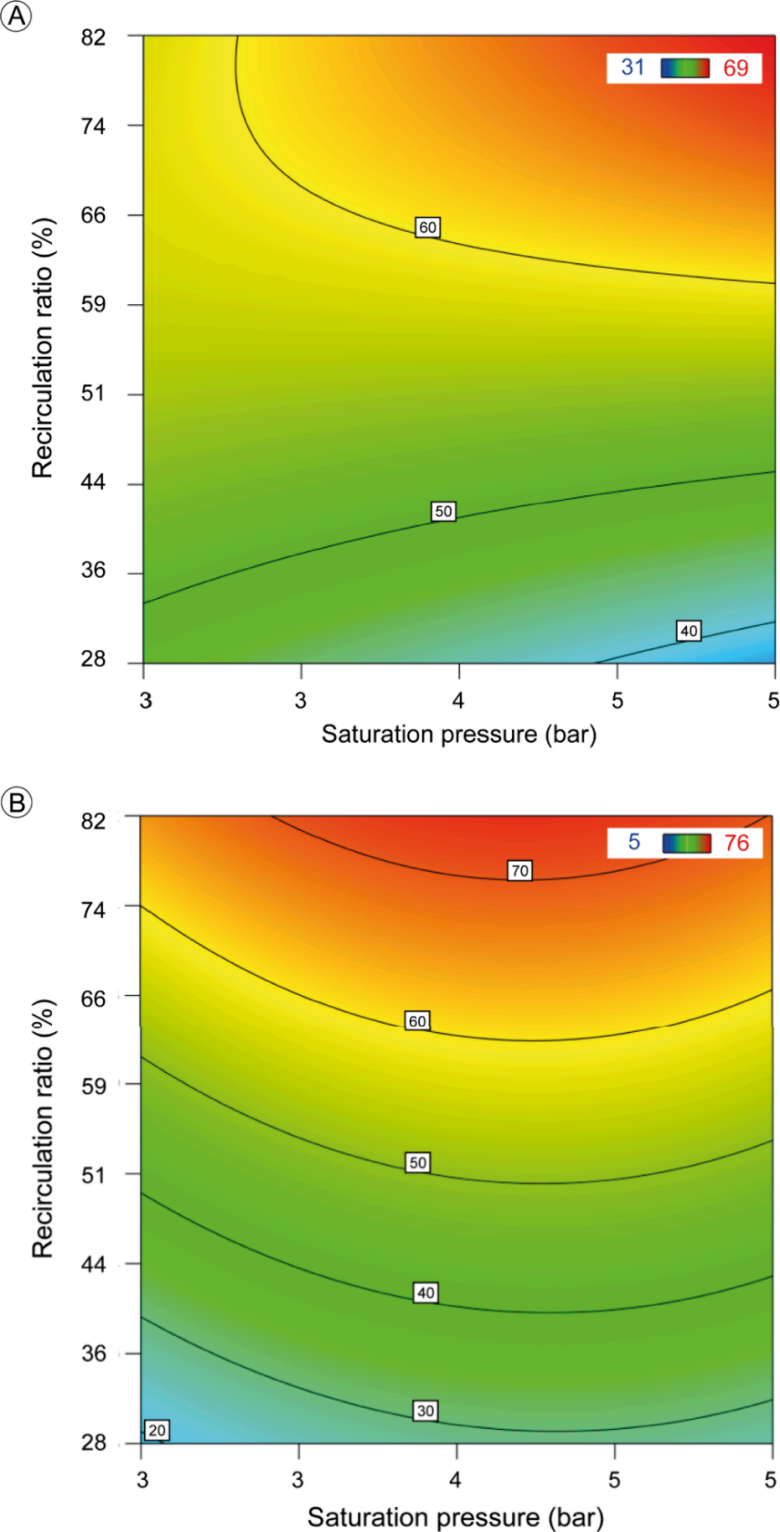
Contour diagrams using calcium chloride to remove turbidity (A) and UV_254_ compounds (B), with the optimum flotation time set at 30 min for both cases.

**Figure 2:**
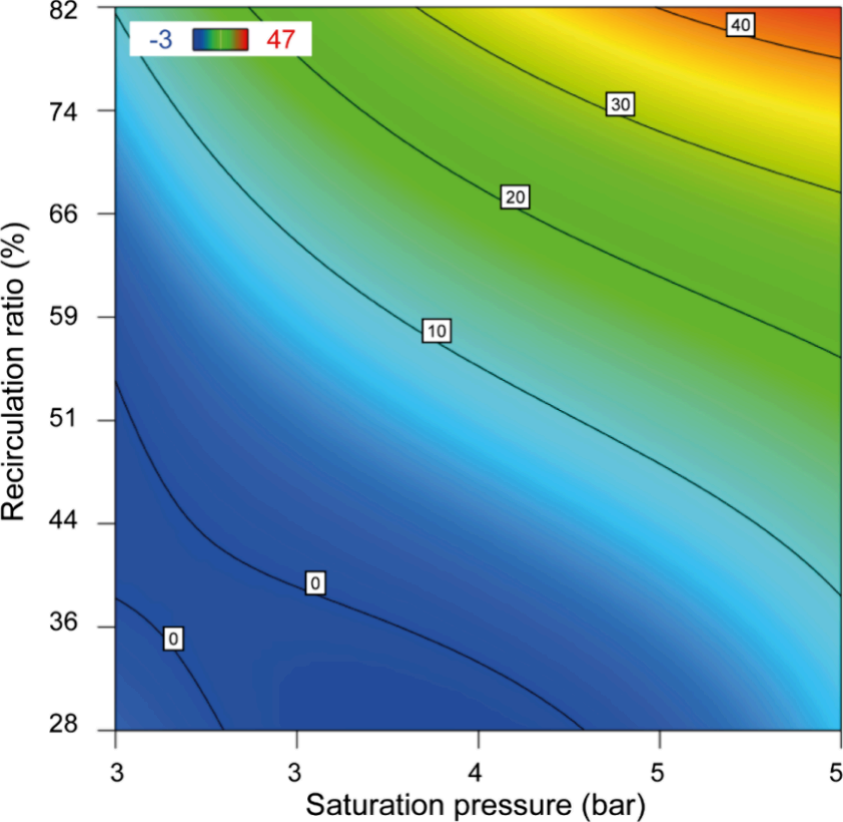
Contour diagram in the absence of calcium chloride for turbidity removal, with the optimum flotation time set at 36 min.

Looking at Figure 1A, it can be seen that the maximum turbidity removal was achieved under saturation pressures above 4.5 bar and recirculation ratios above 70%. This greater removal is also related to calcium chloride, as this salt possibly forms denser and more stable flocs. The saturation pressure influences the properties of bubbles generated in the DAF. In this study, pressures ranging from 2 to 6 bar were applied to understand the effect of pressure on the process. By increasing the saturation pressure, the solubility of air in water also increases, resulting in greater turbidity removal during the process.^20^

Calcium ions (Ca^2+^) are more effective under conditions of high turbidity because more concentrated suspensions are more affected by the action of these ions. This characteristic is advantageous for CPW, which has high turbidity and benefits from the role of calcium in the clarification process.^21^

Lee et al.^22^ studied the use of calcium chloride combined with polyacrylamide (CaCl_2_–PAM) to remove turbidity from kaolin. The authors observed more accurate results in acidic media using a low dose of the compound, varying between 2 and 3 mg L^–1^. This result is consistent with the findings of this study, as there was no change in the pH of the CPW and the amount of calcium chloride used was 1.5 g L^–1^. According to the authors, calcium chloride neutralized the charge on the surface of the wastewater, facilitating particle aggregation.

Analyzing Figure 1B, it can be seen that the greatest removal of UV_254_ compounds, with an efficiency of 70%, occurred at a pressure above 3 bar. It can also be seen that it is necessary for the recirculation ratio to be higher than 80%, indicating that a higher recirculation ratio favors the removal of these compounds. The removal of 70% highlights the contribution of calcium chloride in removing the organic compounds present in the CPW.

CPW has a high value for UV_254_ compounds, which is associated with the presence of aromatic organic matter, characterized by double-bonded ring structures.^23^ The removal of UV_254_ compounds is influenced by the use of divalent salts, such as calcium chloride, because of their ability to intensify the particle aggregation process.

The addition of calcium chloride releases Ca^2+^ ions, which increase the ionic strength of the solution (which already contained a considerable concentration of potassium) and the neutralization of negative charges on the surface of colloids,^24^ promoting to a certain extent the compression of the electric double layer. These mechanisms reduce electrostatic repulsion, favoring particle aggregation and the formation of denser and more stable flocs, thus improving the coagulation-flocculation process.^25^ The resulting flocs are more stable and adhere more efficiently to microbubbles, which facilitates their separation during dissolved air flotation (DAF).^24^

Although the compression of the electric double layer is often considered an important effect of the addition of simple electrolytes, such as CaCl_2_, at low concentrations, this effect may be insufficient to significantly alter the ionic strength of the solution. In this way, the improvement observed in the treatment performance in this research likely results from the combined action of multiple coagulation mechanisms. In this context, Davis and Edwards^26^ demonstrated that calcium can contribute to the coagulation of waters containing natural organic matter, primarily through charge neutralization. The interaction of Ca^2+^ with functional groups in the organic matter reduces the negative charge and decreases the competition with ferric ions, increasing their availability for coagulation. Considering that only CaCl_2_ was used in this study, it is likely that similar mechanisms contributed to the results, highlighting the role of calcium in charge neutralization.

The zeta potential results indicate that the addition of CaCl_2_ to CPW promoted the partial neutralization of the surface charges of the colloidal particles. The raw effluent exhibited a highly negative value (−17.55 mV), indicating strong electrostatic repulsion between the particles. After the addition of CaCl_2_, the zeta potential increased to −10.3 mV, indicating partial charge neutralization and reduced repulsion, allowing closer approach of the particles. Under more acidic conditions (pH < 2), the zeta potential approached neutrality (+0.15 mV), evidencing almost complete neutralization and the emergence of attractive forces that promote aggregation.

In addition to reducing electrostatic repulsion through compression of the electric double layer, studies by Wu et al.^27^ show that Ca^2+^ can complex with functional groups in the organic matter, promoting partial neutralization of negative charges. Although this effect does not bring the system to the isoelectric point, it enhances interactions between colloids and contributes to the formation of more stable flocs, even in the presence of residual electrostatic repulsion.

Thus, the role of calcium in coagulation should be interpreted as a result of complementary mechanisms. In addition to charge neutralization, Ca^2+^ ions may simultaneously bind to negative functional groups in distinct colloidal particles, forming interparticle bridges that promote aggregation and increase floc stability. The adsorption of Ca^2+^ to colloidal surfaces enhances these interactions and intensifies the effects of double layer compression, favoring the formation of denser and more stable flocs and, consequently, greater removal of suspended solids, resulting in reduced turbidity^28^ and compounds responsible for absorption at 254 nm.^29^

The saturation pressure and recirculation ratio interacted with the flocs formed by the action of calcium chloride. This effect influences the efficiency of DAF in removing the organic compounds responsible for UV_254_ absorption. The lower pressure required to remove these compounds compared with turbidity contributes to less fragmentation of the flocs formed, making intact flocs more efficient at capturing organic compounds. The high recirculation ratio, in turn, increases the amount of water recirculated in the system, favoring contact between the suspended particles and microbubbles, which facilitates the removal of UV_254_ compounds.

A comparison of Figure 2 and Figure 1A shows that without the use of calcium chloride, DAF was still able to remove 40% of the turbidity. This emphasizes the importance of adjusting the process parameters, which contribute to the removal of suspended solids even in the absence of salt.

When analyzing the three contour graphs, it can be observed that the recirculation ratio is essential for the operation of the DAF. This parameter controls the number of air bubbles produced in the flotation column. These air bubbles bind to suspended particles and other contaminants in water, helping to remove them.^30^

It has been observed that a high recirculation ratio is necessary for the removal of turbidity and UV_254_ compounds. This is because high values of this parameter help to maintain the balance of the DAF process, as all these factors influence the balance between flocs and bubbles.^31^

The saturation pressure is the key to removing pollutants. This parameter has a direct influence on the amount of air dissolved in water; it should be noted that higher pressures allow for greater dissolution of air, which results in the formation of smaller bubbles during flotation. Smaller bubbles have a larger surface area and are more effective at capturing suspended particles. Therefore, adjusting the pressure in the saturator is an essential factor as it allows the amount of air released into the system to be regulated. In DAF, this contributes to the elevation of light suspended solids, forming a sludge layer that prevents these solids from leaving the treated water, optimizing the turbidity removal process.^32^

Thus, both the recirculation ratio and saturation pressure are essential parameters in the DAF process. An adequate saturation pressure, combined with a high recirculation ratio, favors the formation of microbubbles that adhere to the flocs, facilitating their rapid rise and removal.^33^

In addition, the flotation time plays an essential role, as it relates to the period required for an effective interaction between the microbubbles and flocs. This ensures that all flocs reach the appropriate contact zone and attach themselves to the microbubbles, promoting an effective and efficient collision that results in the separation and removal of pollutants.^31^

It should be noted that the results indicated that the optimum conditions for removing turbidity and UV_254_ compounds in the presence of calcium chloride were a saturation pressure of 5 bar, a recirculation ratio of 80%, and a flotation time of 30 min. Without the addition of salt, the efficiency of the process was reduced, requiring a longer flotation time (36 min) to achieve 40% removal of turbidity without a significant removal of UV_254_ compounds.

The optimum point with calcium chloride (5 bar, 80%, and 30 min) was analyzed, in addition to evaluating turbidity and UV_254_ compounds. Under these conditions, the treatment promoted the removal of 50% of the chemical oxygen demand (COD), 30.3% of total solids, 21.3% of fixed solids, and 32.4% of total volatile solids. The calcium concentration increased from 14.0 to 64.6 mg L^–1^ due to the addition of CaCl_2_, while the potassium concentration varied from 151.5 to 179.5 mg L^–1^.

In addition to the process performance, it is important to consider the fate of the generated sludge. As it does not contain metals commonly used as coagulants, such as aluminum or iron, this material could have the potential for agricultural use, which would reduce possible environmental restrictions. However, a more comprehensive assessment, considering not only sludge disposal but also the entire treatment process, would be necessary in future studies using a life cycle assessment (LCA) approach, allowing a more complete evaluation of the potential environmental impacts.

## 4 Conclusions

Calcium chloride plays an important role in the coagulation-flocculation process, followed by dissolved air flotation. The addition of this salt resulted in superior removal of turbidity and UV_254_ compounds since no significant removal of this last parameter occurred without the use of salt.

The recirculation ratio and saturation pressure are important variables in the DAF process because they determine the quantity and characteristics of the bubbles produced in the process, thereby influencing the removal efficiency of the pollutant particles.

The optimum ranges with the use of calcium chloride were a pressure of 5 bar, a recirculation ratio of 80%, and a flotation time of 30 min, achieving 65% removal of turbidity and 70% of UV_254_ compounds. In the absence of calcium chloride, the optimum conditions were the same for pressure and recirculation ratio (5 bar and 80%), but the time required was 36 min, achieving only 40% turbidity removal.
